# Influence of ligation method on friction resistance of lingual brackets with different second-order angulations: an in vitro study

**DOI:** 10.1590/2177-6709.21.4.034-040.oar

**Published:** 2016

**Authors:** Graziane Olímpio Pereira, Carla Maria Melleiro Gimenez, Lucas Prieto, Marcos Gabriel do Lago Prieto, Roberta Tarkany Basting

**Affiliations:** 1 Research Assistant, São Leopoldo Mandic, Department of Dental Material and Restorative Dentistry, School of Dentistry and Research Institute, Campinas, São Paulo, Brazil.; 2 Professor, Universidade de Araras (UNIARARAS), Department of Orthodontics, Araras, São Paulo Brazil. Universidade Estadual Paulista (FOA-UNESP), School of Dentistry, Department of Orthodontics, Araraquara, São Paulo, Brazil.; 3 Private practice, Campo Grande, Mato Grosso do Sul, Brazil.; 4 Professor, São Leopoldo Mandic, Department of Dental Material and Restorative Dentistry, School of Dentistry and Research Institute, Campinas, São Paulo, Brazil.

**Keywords:** Friction, Ligation, Orthodontic brackets.

## Abstract

**Objective:**

To evaluate stainless steel archwire static friction in active and passive self-ligating lingual and conventional brackets with second-order angulations.

**Methods:**

Two conventional lingual brackets for canines (STb light/Ormco; PSWb/Tecnident), and two self-ligating brackets, one active (In-Ovation L/GAC) and the other passive (3D/ Forestadent), were evaluated. A stainless steel archwire was used at 0°, 3° and 5° angulations. Metal ligatures, conventional elastic ligatures, and low friction elastic ligatures were also tested. A universal testing machine applied friction between brackets and wires, simulating sliding mechanics, to produce 2-mm sliding at 3 mm/minute speed.

**Results:**

Two-way analysis of variance demonstrated a significant effect of the interaction between brackets and angulations (*p* < 0.001). Tukey test indicated that the highest frictional resistance values were observed at 5° angulation for In-Ovation L, PSWb bracket with non conventional ligature, and STb bracket with metal ligature. As for 3D, PSWb with conventional or metal ligatures, and STb brackets with non conventional ligature, showed significantly lower static frictional resistance with 0° angulation. At 0° angulation, STb brackets with metal ties, In-Ovation L brackets and 3D brackets had the lowest frictional resistance.

**Conclusions:**

As the angulation increased from 0° to 3°, static friction resistance increased. When angulation increased from 3° to 5°, static friction resistance increased or remained the same. Self-ligating 3D and In-Ovation L brackets, as well as conventional STb brackets, seem to be the best option when sliding mechanics is used to perform lingual orthodontic treatment.

## RESUMO

**Objetivo:**

avaliar o atrito estático de fios de aço inoxidável em braquetes linguais autoligáveis ativos e passivos, e braquetes linguais ligados convencionalmente, com angulações de segunda ordem.

**Métodos:**

dois tipos de braquetes linguais convencionais para caninos (STb light, Ormco, e PSWb, Tecnident) e dois tipos de braquetes autoligáveis, um ativo (In-Ovation L, GAC) e outro passivo (3D, Forestadent), foram avaliados. Um fio de aço inoxidável com angulações de 0°, 3° e 5° foi utilizado. Ligaduras metálicas, elásticas convencionais e ligaduras elásticas de baixo atrito também foram testadas. Uma máquina de ensaio universal causou atrito entre os braquetes e os fios, simulando uma mecânica de deslizamento de 2mm, a uma velocidade de 3mm/minuto.

**Resultados:**

a análise de variância de dois níveis demonstrou haver um efeito significativo resultante da interação entre braquetes e angulações (*p* < 0,001). O teste de Tukey indicou que os valores mais altos de resistência ao atrito foram observados com uma angulação de 5° no braquete In-Ovation L, e no braquete STb com ligadura metálica. Já os braquetes 3D e In-Ovation L, e os braquetes STb com ligadura não convencional apresentaram valores de resistência ao atrito significativamente baixos com uma angulação de 0°. Com angulação de 0°, os braquetes STb com ligaduras metálicas, braquetes In-Ovation L e braquetes 3D apresentaram os mais baixos valores de resistência ao atrito.

**Conclusões:**

à medida que a angulação aumentou de 0° para 3°, a resistência ao atrito estático também aumentou. À medida que a angulação aumentou de 3° para 5°, a resistência ao atrito estático também aumentou ou continuou a mesma. Os braquetes autoligáveis 3D e In-Ovation L, assim como os braquetes convencionais STb, parecem ser as melhores opções quando a mecânica de deslizamento é usada no tratamento ortodôntico lingual.

## INTRODUCTION

Lingual brackets are different from labial brackets in regard to configuration and clinical aspects. Specifically, conventional lingual brackets are smaller sized to increase patient's comfort and improve oral hygiene.^1^ Almost all lingual brackets are single and have narrower mesiodistal width than buccal brackets because of their anatomical limitations, and because they were projected to provide greater interbracket distance, despite being more susceptible to tipping under traction force.^2^ Mechanics work by means of sliding and have the advantage of minimizing the time of closing arch gaps and of reducing the number of activations; however, if teeth are not leveled properly, the increased friction between arches and brackets can generate unexpected dental movements and greater anchorage loss.^2^

Some factors influencing friction resistance are related to the material composing brackets and wires, surface conditions of arches and bracket slot, archwire cross-section, torque at the wire-bracket interface, bonding strength, use of self-ligating brackets, interbracket distance, presence of saliva and influence of oral functions.^3^ *In vitro* studies have evaluated friction resistance among different alloys and wire calibers by means of several ligation methods and material of buccal orthodontic brackets with alterations in angulation, using models with one, three, five and ten brackets, and typodonts to simulate different situations.^4^ ^-^ ^10^ However, few studies on friction produced by lingual brackets have been published.^2^ ^,^ ^11^ ^,^ ^12^

When the angle between the bracket and the arch (second-order angle) increases, frictional resistance (more specifically, binding) appears to increase quickly, and even more quickly beyond the critical contact angle.^2^ If the arch does not bend (i.e., deform elastically), the angle will not increase beyond the critical contact angle, but sometimes there is sliding resistance and some amount of retraction force is lost.^2^ ^,^ ^11^ In this context, it becomes interesting to evaluate static friction resistance of different lingual bracket types (conventional and self-ligating) at different angulations.

## MATERIAL AND METHODS

**Figure 1:**
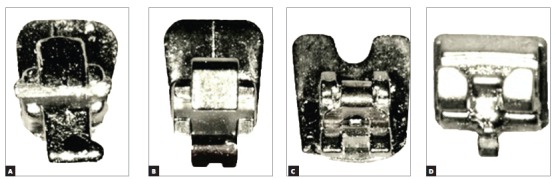
Brackets used in the study: A) PSWb (Tecnident, São Carlos/SP, Brazil); B) STb light (Ormco, USA); C) In-Ovation L (GAC, USA); D) 3D (Forestadent, Germany).

The experimental units were composed of stainless steel brackets of different commercial brands evaluated with a single-diameter rectangular wire submitted to friction at different angles (n = 5).^2^ ^,^ ^9^ The material used in the experimental units and their respective characteristics and manufacturers are shown in Table 1. Brackets used are shown in Figure 1.

**Table 1:**
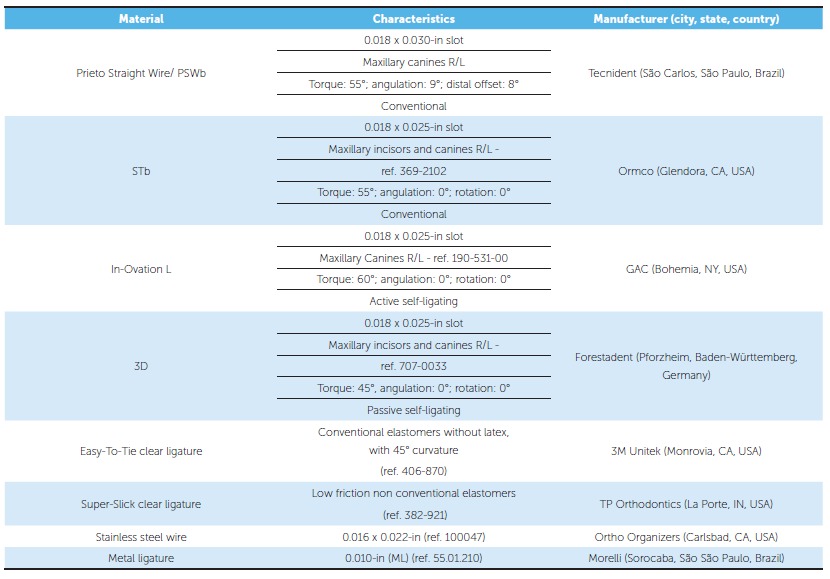
Characteristics of material used in the experiment.

**Figure 2:**
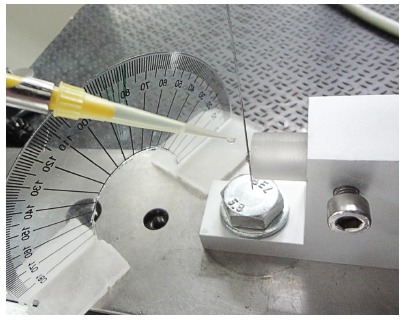
Bracket positioned for the friction test in the device, and application of artificial saliva for lubrication.

Acrylic cylindrical devices were developed for bracket bonding and positioning of angulations to conduct the friction resistance tests. At one end of the device, an L-shaped steel key was manufactured and inserted through a mechanical lathe to ensure the key would not become dislocated within the acrylic device when positioning the second-order angulation. At the other end, bracket bonding was performed for subsequent evaluation. The devices were engaged in a prefabricated metal apparatus screwed onto the base of the universal testing machine (Emic DL 2000, São José dos Pinhais, Paraná, Brazil) in order to carry out the friction trials (Fig 2). For evaluation of bracket positioning in second-order angulation, a plastic Protractor (KJIN, plastic, 180 degrees, Shenzhen, Guangdong, China) was adapted to the metal apparatus to measure the angles (Fig 2). 

The metal wire (0.016 x 0.022-in) (Ortho Organizers) to be pulled was cut 10-cm in length and used as a guide to standardize the positioning of the bracket bonded to the acrylic device. The following bonding procedure was used. Brackets were inserted 10 mm from one end of the wire, while the other end of the wire was inserted 10 mm into the load cell coupled to the universal testing machine for standardization purposes. In doing so, the face of the bonded bracket remained parallel to the edge of the acrylic device, so as to prevent interference from torque and angulation. Fast curing glue (Super Bonder GEL, Henkel, Itapevi, SP, Brazil) was applied to the base of the bracket already attached to the wire in the load cell, according to the connection method to be studied. The metal apparatus screw holding the test specimen was loosened and then inserted tightly against the base of the bracket, taking care not to alter anything, and to retain the bracket-wire set in a "passive configuration."

After bonding each bracket to be tested, the brackets were tied to the wire with different types of ligatures: conventional brackets (STb, PSWb) were tied with conventional ligatures (Easy-To-Tie), non conventional were tied with low-friction (Super Slick) and metal ligatures, and the clip on the self-ligating active (In-Ovation L) and passive (3D) lingual brackets set was locked into place.

Friction resistance tests were performed at room temperature (24 °C) by applying a drop of artificial saliva^13^ (3.5 g porcine mucin; 2 g xylitol; 100 mg metilparabene; 50 mg EDTA; 2 mg benzalkonium chloride; 0.42 mg sodium fluoride; 100 ml aqueous solution) to simulate lubrication of the oral environment. Before starting each test, artificial saliva was applied with a micropipette (HTL Labmate, Daniszewska, Warsaw, Poland) by dripping the solution into the slot of the bracket (Fig 2).

The friction resistance test was performed five times for each one of the established angulations (0°, 3° and 5°). At each repetition, elastomeric and metal ligatures were replaced on conventional brackets. In testing the self-ligating brackets, the machine was repositioned to the initial position, and the clip was opened and closed. Each time the test was performed, artificial saliva was placed in the slot of the bracket to be tested, and the whole bracket-wire set was wiped with cotton soaked in 70% alcohol. 

Initially, the tests were carried out with 0° angulation for a given type of bracket. Subsequently, a new bracket-wire set was positioned in the acrylic device and the load cell, respectively, in accordance with the procedures previously described. To position the bracket in the desired angulation, the screw of the metal apparatus that positions the device was loosened with the appropriate key. The L-shaped steel key of the acrylic device was rotated manually to reach the desired angulation of the bracket-wire set, at 3° or 5°, using a protractor as reference. The bracket-wire set was repositioned at 0° angulation before performing each 3° and 5° angulation test.

The universal testing machine was programmed to pull the wire at a speed of 3 mm per minute, with 2 mm of wire displacement by the bracket slot. Friction resistance generated during wire movement was determined with a load cell of 50 N coupled to a universal testing machine. The universal testing machine and a software application (Tesc version 3.01, São José dos Pinhais, Paraná, Brazil) were used to apply sliding and friction resistance between brackets and wire. Canine distal sliding resistance was simulated from the right side of a 0.016 x 0.022-in wire in a previously aligned arch.

Before data analyses, normality of distribution and homogeneity of variance of values were checked by means of Shapiro-Wilk and Levene tests, and so was the presence of discrepant data. Normal distribution of data was observed and two-way analysis of variance was employed. Tukey test was carried out to perform multiple comparisons. SPSS software 20 (SPSS Inc., Chicago, IL, USA) was used to perform statistical calculations, adopting a significance level of 5%.

## RESULTS

The two-way analysis of variance showed a significant effect of the interaction between brackets and angle factors (*p* < 0.001). Tukey test showed that there was no significant difference in static friction generated by the evaluated brackets-ligatures for the test condition without angulation (0°).

In regard to all angulations (Table 2), conventional STb bracket with conventional ligature presented significantly lower statistical friction than all the other groups represented by bracket-ligatures, with the exception of groups in which STb and bracket metal ligature were used. This set, in turn, provided no friction force different from that observed with the Active In-Ovation L self-ligating bracket. The friction force measured with this last bracket did not differ significantly from that measured for the STb bracket associated with unconventional ligature. When the conventional PSWb system was used with the metal ligature, it also showed no significantly different friction from that observed with the active self-ligating bracket (In-Ovation L). The passive self-ligating system (3D) generated friction values that were not significantly different from those observed with the active self-ligating bracket system (In-Ovation L). The conventional PSWb system associated with conventional ligation resulted in statistically higher friction, compared with the passive self-ligating bracket (3D).

**Table 2:** Mean and standard deviations in gf of static friction force, according to bracket type and angulation.

Bracket - Ligature	0^o^	3^o^	5^o^
3D	18 ± 4 ^Aa^	408 ± 38 ^CDb^	516 ± 25 ^ABCb^
In-Ovation L	10 ± 7 ^Aa^	292 ± 87 ^BCb^	682 ± 110 ^Dc^
PSWb - CL	56 ± 5 ^Aa^	610 ± 139 ^Eb^	488 ± 75 ^ABCb^
PSWb - ML	120 ± 66 ^Aa^	328 ± 131 ^Cb^	376 ± 64 ^Ab^
PSWb - NCL	46 ± 22 ^Aa^	476 ± 35 ^DEb^	684 ± 47 ^Dc^
STb - CL	30 ± 7 ^Aa^	132 ± 55 ^Aa^	530 ± 45 ^BCb^
STb - ML	0 ± 0 ^Aa^	164 ± 48 ^ABb^	632 ± 86 ^CDc^
STb - NCL	90 ± 12 ^Aa^	322 ± 41 ^Cb^	412 ± 58 ^ABb^

CL: Conventional Ligature; ML: Metal Ligature; NCL: Non Conventional Low Friction Ligature. Means followed by different superscript capital letters indicate significant difference among brackets-ligatures, considering each angulation individually (comparisons within each column). Mean followed by distinct superscript lowercase letters indicate significant difference among angles, considering each individual bracket-ligature (comparisons within each line).

Under 5° angulation, there was no significant difference in friction force values provided by PSWb brackets with metal or conventional ligature, STb brackets with unconventional ligature and passive 3D self-ligating brackets. The highest static friction values were recorded for In-Ovation L brackets and associated non conventional PSWb ligature, which, in turn, did not differ significantly from STb bracket with metal ligature.

Tukey test indicated that the least friction force was measured in the absence of angulation, whereas the highest values were observed in 5° angulation for the active self-ligating bracket (In-Ovation L), conventional PSWb with unconventional ligature, and STb with metal ligature. Static friction force for the passive self-ligating system (3D), PSWb conventional brackets with conventional or metal ligatures, and STb brackets associated with an unconventional ligature, were significantly lower at an angle of 0°, but there was no significant difference between friction values generated with 3° and 5° angulations.

Only STb bracket with metal ligature showed no difference in friction force at 0° and 3° angles. The highest friction force for the STb bracket was observed in 5° angulation.

## DISCUSSION

As regards the method of bracket ligation, the present study showed no statistically significant differences at 0° angulation. However, several studies with labial brackets and the few existing studies with lingual brackets showed that increasing reduction or friction depended on the ligation method using passive or active self-ligating brackets, conventional or unconventional elastomeric ligatures, metal ligature types, and different wires alloys and gauges.^5^ ^,^ ^8^ ^-^ ^12^ ^,^ ^14^ ^-^ ^18^

Super Slick ligatures have been thought to reduce friction because of their Metafix coating which reduces friction and adhesion of residues and plaque.^19^ However, in the present research, these ligatures did not cause less friction, corroborating the findings of other studies^14^ ^,^ ^20^ ^,^ ^21^ ^,^ ^22^ which evaluated different kinds of elastic ligatures, including Easy-to-Tie and Super Slick. The latter showed the least resistance to friction, while the former showed the smallest values; however, metal ligatures and self-ligating methods were not compared. On the other hand, other studies^4^ ^,^ ^20^ ^,^ ^23^ showed greater friction than that of self-ligating brackets and metal ligatures.

Other studies have shown that elastic Super Slick reduced friction resistance, compared with other conventional elastic ligatures, including Easy-to-Tie,^4^ ^,^ ^24^ not corroborating the results of this study. Hain et al^4^ observed that Super Slick ligatures increased friction by 80% when not immersed in human saliva. For this reason, artificial saliva lubrication was used in this study. Leanderand and Kumar^19^ reported that Metafix is a hydrophobic coating and becomes slippery in the presence of water or saliva, thus reducing friction.

Thorstenson and Kusy^25^ reported the importance of observing the critical angle, i.e., the angle in which the wire touches the opposite corners of the bracket. At this point, friction increases significantly with any type of bracket and wire, and when the arch does not deform beyond the critical angle (known as binding), sliding resistance occurs and some portion of the retraction force is lost. The present study showed that increased angulation led to increased resistance to friction, corroborating the findings of other authors who have studied labial and lingual braces.^2^ ^,^ ^11^ ^,^ ^25^. The choice of 0°, 3° and 5° angulations for this research protocol was based on previous studies available in the literature.^2^ ^,^ ^11^ ^,^ ^25^ More specifically, the findings of Park et al^2^ described the critical angle as ranging between 1° and 3° when using lingual braces in second-order angulations with 0.018 x 0.025-in slots and coupled to 0.016 x 0.022-in stainless steel wires. Therefore, angulations greater than 5° were not needed to evaluate canine retraction. In addition, orthodontic dental movement is not continuous and linear, but rather dynamic and discontinuous; that is why second-order angulations must be evaluated.^2^ ^,^ ^11^ ^,^ ^25^

Ortan et al^11^ reported that the ideal wire for partial canine retraction would be a 0.016 x 0.016-in stainless steel wire, and the ideal wire for mass retraction would be a 0.016 x 0.022-in stainless steel wire. Partial canine retraction becomes infeasible in lingual orthodontic treatment because patient's esthetics would be compromised, and the patient opted for this mode of treatment due to its good esthetic results. Therefore, mass retraction would be the best choice. However, no *in vitro* model has been reported up to date, which could reproduce this research performed by retracting the six anterior teeth.^2^ ^,^ ^11^

For maximum anchorage cases, low-friction brackets seem to be the most effective alternative in posterior segments when sliding mechanics is used.^11^ In lingual Orthodontics, low-friction brackets may increase the risk of mesiobuccal molar rotation, distobuccal canine rotation and expansion of the arch, causing a transverse bowing effect.^2^ ^,^ ^11^ The brackets with elastic ligatures and PSWb brackets with metal ligature presented the highest resistance to static friction, probably due to their deep slot design, and may offer clinical significance. However, friction-related studies on lingual brackets are still too few to make comparative conclusions,^2^ ^,^ ^12^ although Lalithapriya et al^12^ showed that self-ligating brackets may not be beneficial in reducing friction during en-mass retraction due to their interactive clip type.

In 3° angulation, STb brackets with conventional ligatures had the least resistance to friction and showed no statistically significant difference between 0° and 3° angulations, a situation in which STb bracket with metal ligature performed similarly. This can be explained by the variation in the critical contact angle between 1° and 3° with 0.016 x 0.022-in wires.^2^ Despite the reduced static friction resistance of this group, in comparison with the other groups, this cannot be considered a positive factor for lingual brackets, since the same feature also acts as a negative factor, owing to reduced control offered by a single bracket.^2^ ^,^ ^11^ In general, resistance to static friction increased significantly when the angulation increased to 3° and 5°, corroborating previous studies.^2^ ^,^ ^11^ ^,^ ^25^

In lingual orthodontic treatment in which lower resistance to friction is required, self-ligating brackets (3D, In-Ovation L) and conventional brackets (STb) with metal ligatures appear to be the best treatment option. Although this study has some limitations due to the characteristics of an *in vitro* trial, such as over controlled variables, number of specimens per group and differences in slot designs among brackets, the results of this current research could influence and guide orthodontists who prefer to use lingual brackets in their clinical practice. The studies by Ortan et al^11^ found lower friction force with In-Ovation L brackets, compared with the conventional brackets evaluated in this research, corroborating the findings of our research. Self-ligating brackets have the advantage of providing shorter chair time, in comparison with metal ligatures, because they are easier to handle and minimize the risk of a metallic washer tip escaping during eating or brushing, an event that could hurt patient's tongue.^11^ Although PSWb brackets have a 0.018 x 0.030-in slot, they showed no reduced friction resistance related to greater depth, in comparison with the other brackets evaluated in this experiment, probably due to some irregularities in the slot, which could influence friction. However, scanning electron microscopic evaluations should be taken to confirm this hypothesis. Ortan et al^11^ evaluated the slot dimensions of the brackets studied by means of scanning electronic microscopy and concluded that the values reported by the manufacturers did not match those of the research results, and that all brackets had larger dimensions. The significance of these findings is that lower friction can be induced by a larger slot size, and this could cause problems of second and third orders, by compromising torque and rotation control.^11^ Therefore, the use of 0.022-in slot brackets could also provide lower friction resistance when using 0° angulation due to a large slot design that may result in a gap between the archwire and the bracket; however, an increase in angulation produces higher friction.

## CONCLUSIONS

In spite of the limitations of this *in vitro* study, some conclusions can be drawn, as follows:

- When angulation increased from 0° to 3°, static friction resistance increased. When angulation increased from 3° to 5 °, static friction resistance increased or remained the same.
- Self-ligating 3D and In-Ovation L brackets, as well as conventional STb brackets, seem to be the best option when sliding mechanics is used to perform lingual orthodontic treatment.
